# Cotton-top tamarins (*Saguinus oedipus*) can serve as definitive hosts of the rodent metastrongyloid parasite *Angiostrongylus dujardini* and develop fatal infections

**DOI:** 10.1016/j.ijppaw.2026.101256

**Published:** 2026-06-19

**Authors:** Seraina L. Meister, Christian Wenker, Michael Rüttener, Gastón Moré, Walter Basso

**Affiliations:** a Institute of Animal Pathology, Department of Infectious Diseases and Pathobiology, Vetsuisse Faculty, University of Bern, Länggassstrasse 122, Bern, CH-3012, Switzerland; b Zoo Basel, Binningerstrasse 40, Basel, CH-4054, Switzerland; c Institute of Parasitology, Department of Infectious Diseases and Pathobiology, Vetsuisse Faculty, University of Bern, Länggassstrasse 122, Bern, CH-3012, Switzerland

**Keywords:** *Angiostrongylus*, Nematode, Callitrichidae, Zoo, PCR, ITS2

## Abstract

*Angiostrongylus dujardini* is a heteroxenous metastrongyloid nematode that primarily inhabits the pulmonary arteries and the right heart of murid and cricetid rodents and uses gastropods as intermediate hosts. Although not considered highly pathogenic for rodents, this parasite has been reported to cause death in callitrichid monkeys, suricates and a psittaciform bird under human care. In contrast to rodents, shedding of first-stage larvae (L1) in the faeces has not been documented in these species; consequently, they have been regarded as accidental hosts.

This study reports fatal *A. dujardini* infections in seven cotton-top tamarins (*Saguinus oedipus*) from a Swiss zoological garden between 2005 and 2024, and L1 shedding in one of the individuals. Five monkeys were found dead without prior clinical signs, while two showed weakness shortly before death. Gross pathological findings at necropsy included dark red pulmonary discoloration, lung nodules, intrathoracic effusion, right heart dilation and subepicardial haemorrhages. Histopathological examination revealed multifocal collection of thin-walled, embryonated nematode eggs at variable stages of development, along with fully developed larvae within the lumina of alveoli and pulmonary vessels, surrounded by granulomatous inflammation, consistent with granulomatous pneumonia and endarteritis in all cases. In the most recent fatal case, *A. dujardini* L1 were detected in the faeces, providing evidence that *S. oedipus* can act as a definitive host of this parasite, capable of environmental contamination. The diagnosis of *A. dujardini* infection was achieved by histopathological and molecular investigations of lung tissues and faecal larvae. This is the first report of *A. dujardini* infection with larval shedding in a non-human primate, confirming their role as potential definitive hosts, and underlining the need for heightened parasitological surveillance in captive primate populations.

## Highlights

• *Saguinus oedipus* can act as definitive host of *Angiostrongylus dujardini*.
• First evidence of *Angiostrongylus dujardini* larval shedding in a non-human primate.
• *Angiostrongylus dujardini* exhibits marked pathogenic potential in *Saguinus oedipus*.
• Most individuals died unexpectedly without prior clinical signs.
• Affected animals were 4 months to 6.5 years old at the time of death.

## Graphical abstract

**Figure:**
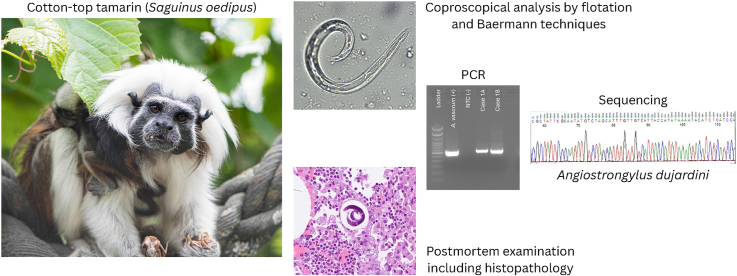

## 1 Introduction

Cardiopulmonary nematodes of the genus *Angiostrongylus* (superfamily *Metastrongyloidea*) are known for their complex life cycles and their capacity to cause significant pathology and disease in a variety of mammalian hosts including humans. Like other species within the genus, *Angiostrongylus* (syn. *Parastrongylus*) *dujardini* follows an indirect life cycle involving aquatic and terrestrial gastropod intermediate hosts (Doby et al., 1971) in which first-stage larvae (L1) develop into infective third-stage larvae (L3). The definitive host becomes infected through ingestion of these infected molluscs or, occasionally, paratenic hosts. Adult worms reside in the pulmonary arteries and right heart, where females lay eggs that embolize in the pulmonary capillaries. First-stage larvae develop within these eggs, hatch in the lungs, migrate up the airways, are swallowed, and are subsequently shed in the faeces after a prepatent period of 24–26 days (Spratt, 2015).

This parasitic nematode, historically associated with rodents, has increasingly drawn attention due to its capacity to infect a range of non-rodent hosts in zoological settings. Although originally described from wild murid and cricetid rodents in Europe (Dróżdż and Doby, 1970; Doby et al., 1971; Mészáros, 1972; Tenora et al., 1983; Gerrikagoitia et al., 2010; Spratt, 2015), sporadic reports of fatal infections in captive exotic species, especially primates, have raised concerns about the parasite's host range, pathogenicity, and transmission dynamics outside its presumed natural cycle. Infections with *A. dujardini* have been previously recorded in several species of tamarins and marmosets, including cotton-top tamarins (*Saguinus oedipus*), Goeldi's tamarins (*Callimico goeldii*), a white-lipped tamarin (*Saguinus labiatus),* white-headed marmosets (*Callithrix geoffroyi*), a pygmy marmoset (*Callithrix pygmaea*), as well as in suricates (*Suricata suricatta*) (Graille et al., 2015; Eleni et al., 2016) and more recently in a coconut lorikeet (*Trichoglossus haematodus*) (Meister et al., 2024). This recent detection of *A. dujardini* in an avian species marked a significant development, providing the first reported evidence of natural infection in a non-mammalian vertebrate. The finding suggests that the host range of *A. dujardini* may be broader than previously assumed and is not limited to mammals, further complicating the understanding of its transmission dynamics.

The cases in zoo animals were characterized by severe pulmonary lesions and often sudden death, yet their role as definitive host could not be confirmed due to the absence of larval shedding in faeces or lack of testing. As a result, the involvement of these animals in the parasite's life cycle, particularly their potential to contribute to environmental contamination, has remained uncertain.

In primates, especially New World monkeys such as tamarins (Callitrichidae), angiostrongyliasis is typically associated with acute disease and high mortality. The parasite's development in these hosts, however, has been poorly understood, with limited molecular confirmation and no conclusive evidence of a complete life cycle. Understanding whether affected species act as accidental or definitive hosts is critical not only for clarifying epidemiological pathways but also for implementing parasite control in zoological institutions.

The cotton-top tamarin (*Saguinus oedipus*) is a small callitrichid primate endemic to the lowland tropical forests of northwestern Colombia and is currently classified as Critically Endangered on the IUCN Red List (Rodríguez et al., 2021). The remaining wild population is estimated at around 6000–±7000 individuals, with numbers continuing to decline (Savage et al., 2010, 2016; Rodríguez et al., 2021). Major threats include severe habitat destruction due to deforestation for agriculture, mining and urban expansion, as well as illegal capture for the pet trade. Historically, cotton-top tamarins were also heavily exploited for biomedical research, with more than 20,000–30,000 individuals captured and exported for this purpose during the 1960s and early 1970s (Savage et al., 2016, 2023). Currently, there are 415 zoological institutions worldwide keeping this monkey species under human care, including 177 in Europe and five in Switzerland, with the Zoo Basel maintaining the species since 1961 (https://www.zootierliste.de/). Notably, captive populations retain higher genetic diversity than wild populations, making them a valuable *ex situ* resource to enhance wild populations in potential resettlement programs (Savage et al., 1996).

This report presents a retrospective, long-term investigation of fatal *A. dujardini* infections in cotton-top tamarins (*S. oedipus*) from the Swiss Zoo Basel over a 19-year period (2005–2024).

## 2 Materials and methods

### 2.1 Study background and investigation context

At Zoo Basel, a 5.5-year-old female cotton-top tamarin, weighing 470 g, born at this zoo and housed with a male companion in the same enclosure, was found dead in the morning without any prior clinical signs (case 1). No external injuries were noticed. Gross, histopathological and parasitological findings were consistent with a fatal *Angiostrongylus* sp. infection. Based on this case and the fact that a coconut lorikeet from the same institution had recently succumbed to *A. dujardini* infection (Meister et al., 2024), an investigation including retrospective case analyses in cotton-top tamarins was initiated. Faecal samples from the surviving male monkey in the same enclosure were also analysed coproscopically as described below, data on management and diet were recovered, and archived records and histological samples from further monkeys that died during the last 20 years at the Zoo Basel were reviewed. Six additional cotton-top tamarins that died at the zoo with postmortem findings suggestive of lungworm infection were identified (cases 2–7).

### 2.2 Husbandry and diet

The cotton-top tamarins were housed in an indoor enclosure connected to a naturalistic outdoor enclosure located on the green roof of the primate house. Adjacent to their enclosure, but without direct contact, were Bolivian squirrel monkeys (*Saimiri boliviensis*) and Brown woolly monkeys (*Lagothrix lagothricha*). Occasional contact with rodents within the same building could not be excluded. Various wild bird species were present in the outdoor areas of the facility, and terrestrial molluscs are known to occur within and around the outdoor enclosure. The monkeys’ daily diet included vegetables such as fennel, Chinese cabbage, celery, broccoli, kohlrabi, leek, cucumber, tomato, bell pepper, chicory, beetroot, and steamed carrots and sweet potatoes; fruits such as bananas, papaya and grapes; as well as cottage cheese and boiled eggs. Grasshoppers were offered three times per week, and one mouse per animal was provided once per week. Due to previous cases of lungworm infections without a definitive aetiological diagnosis, the tamarins received a monthly antiparasitic treatment using an oral liquid fenbendazole formulation (Panacur 10%, MSD Animal Health, Luzern, Switzerland; 50 mg/kg per os, once daily for five consecutive days) applied via food.

### 2.3 Necropsy and histopathological examination

Necropsy and histopathological examination were performed on all seven cotton-top tamarins at the Institute of Animal Pathology of the University of Bern. Various organs were sampled during necropsy and fixed in 4% buffered formalin for 24 h to seven days. Fixed tissues were processed routinely for histopathological evaluation: formalin-fixed tissues were embedded in paraffin, sectioned at 2–4 μm, mounted on glass slides and stained with haematoxylin and eosin (H&E).

### 2.4 Coproscopical examination

Faecal material was collected from the most recent female individual (case 1) during necropsy and coproscopical analyses were performed using zinc chloride flotation and the Baermann technique. Faeces from the surviving male monkey in the same enclosure were additionally analysed.

### 2.5 DNA extraction

DNA was extracted from 20 μm-thick formalin-fixed and paraffin-embedded (FFPE) sections of lung tissues as previously described (Müller et al., 2003) and, in case 1, additionally from recovered faecal larvae (Quick-DNA Fecal/Soil Microbe Miniprep Kit, Zymo Research, USA), according to the manufacturer's protocols for the commercial kits used.

### 2.6 PCR analysis and sequencing

A PCR targeting the ITS2 plus 5.8 S and 28 S rDNA flanking regions of lungworms was performed on DNA from the faecal larvae (case 1), and lung tissues from all seven monkeys (cases 1–7), and the amplification products were purified and sequenced, proceeding exactly as previously described (Meister et al., 2024). The resulting sequences were aligned using the Geneious software (Geneious Prime® 2023.0.4) and consensus sequences were compared with those in GenBank using the Nucleotide BLAST algorithm (http://blast.ncbi.nlm.nih.gov/Blast.cgi).

## 3 Results

### 3.1 Gross and histopathological findings

At necropsy of case 1, the lungs exhibited multifocal to coalescing areas of reddening and emphysema. Histological examination revealed multifocal to coalescing, prominent granulomas affecting blood vessels, alveoli and interstitium (Fig. 1A–D). These were composed predominantly of epithelioid macrophages and multinucleated giant cells including foreign body-type (Fig. 1E) and Langhans-type (Fig. 1F), along with lymphocytes, plasma cells, and fewer neutrophils and eosinophils. The granulomas were associated with necrotic areas characterized by hypereosinophilia, karyopyknosis, karyorrhexis, karyolysis and accumulation of necrotic cellular debris, admixed with extravascular erythrocytes (haemorrhage). Multifocal accumulations of ovoid, thin-shelled, embryonated eggs at various developmental stages, as well as fully developed nematode larvae with a thin cuticle, were observed within pulmonary arterioles, alveoli, bronchioles, and necrotic foci, consistent with infection by metastrongyloid nematodes (Fig. 1A–D). The walls of multiple small arterioles were multifocally replaced by bands of brightly eosinophilic, amorphous to flocculent material (fibrinoid necrosis) and were partially or completely occluded by fibrin thrombi (Fig. 1A). The tunica intima was multifocally thickened by variable amounts of fibrous connective tissue and inflammatory cells, consistent with chronic proliferative endarteritis. In addition, pulmonary congestion, emphysema, as well as alveolar and perivascular oedema were present. Sections of heart, liver, spleen, and kidney were histologically unremarkable.

**Fig. 1:**
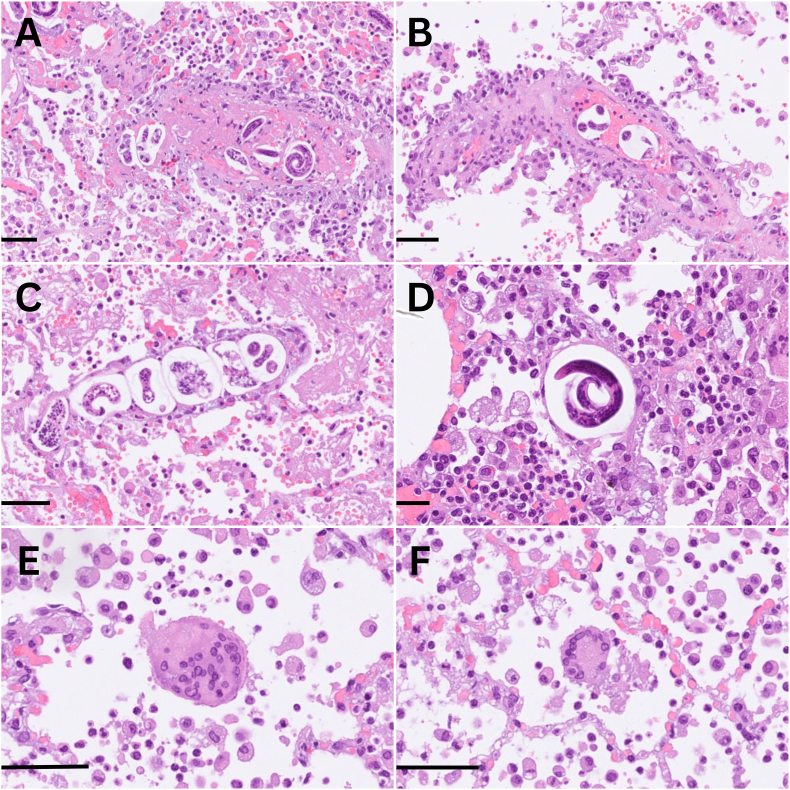
Histopathological lesions in the pulmonary tissue of a female *Saguinus oedipus* (case 1). Light microscopic view of multifocal aggregates of ovoid, thin-walled, embryonated eggs at different developmental stages and nematode larvae. The parasites are located within pulmonary arterioles (A, B), the alveolar lumen and interstitium (C, D). Granulomatous inflammatory response characterized by concentrically arranged epithelioid macrophages, multinucleated giant cells (foreign-body type [E] and Langhans type [F]), lymphocytes, plasma cells, and fewer neutrophils and eosinophils. These findings are consistent with severe, chronic, multifocal granulomatous pneumonia with endarteritis, associated with *Angiostrongylus infection.* Scale bars: 50 μm.

Signalment, clinical history, gross and histopathological findings as well as molecular diagnostic results of all seven cases are summarized in Table 1.

**Table 1:** Summary of seven cases of *Angiostrongylus dujardini* infection in cotton-top tamarins (*Saguinus oedipus*), including animal data and main clinicopathological and molecular findings.

	Case 1	Case 2	Case 3	Case 4	Case 5	Case 6	Case 7
**Voucher No.**	S24_7131	Z05_3257	Z08_1729	Z09_2431	Z09_3210	S12_4873	S12_5041
**Birth location**	Switzerland	Switzerland	Denmark	Switzerland	Switzerland	Switzerland	Switzerland
**Death date**	05.12.2024	14.08.2005	26.04.2008	17.06.2009	16.08.2009	20.10.2012	05.11.2012
**Age**	5 y 5 m	1 y 1 m	6 y 6 m	2 y 3 m	4 m	11 m	2 y 7 m
**Sex**	Female	Female	Female	Male	Female	Male	Female
**Body weight (g)**	470	360	472	N/A	230	400	449
**Clinical history**	Found dead without prior clinical signs	Same as case 1	Gave birth to 2 weak offsprings; one was euthanised, the other died on day 4; mother was in poor condition and died	Same as case 1	Appeared tired the evening before and was found dead the following day	Same as case 1	Same as case 1
**Gross pathological findings**	See text	Thorax: contained 8 ml serosanguineous fluid Lung: same as case 1	Thorax: contained 2 ml serosanguineous fluid Heart: right heart dilation; multifocal subepicardial petechial and ecchymotic haemorrhages Lung: same as case 1 + oedema	Severely autolytic	Lung: scattered 0.5 cm in diameter, white foci of firm consistency in the dorsal aspect	Lung: same as case 1	Lung: same as case 1 + multifocal, 0.5 in diameter, firm nodules
**Histopathological findings**	See text	Lung: same as case 1 + leukocytostasis (predominantly neutrophils) Heart: multiple adult nematodes in the right ventricle; multifocal degenerating cardiomyocytes with focal mineralisation, surrounded by neutrophilic, eosinophilic, and lymphocytic inflammation	Lung: same as case 2 Heart: focal, small focus with neutrophilic infiltration Liver: multifocally centrilobular necrosis (hypoxia)	Same as case 1	Lung: same as case 1 Heart: numerous cross-sections of adult nematodes in the right ventricular lumen	Same as case 1	Same as case 1
**PCR result**	Positive	Negative	Negative	Positive	Positive (weak)	Positive	Positive
**Sequencing result**	*Angiostrongylus dujardin*i	N/A	N/A	*Angiostrongylus dujardini*	N/A	*Angiostrongylus dujardini*	*Angiostrongylus dujardini*
**GenBank accession number**	PZ267713	N/A	N/A	PZ267714	N/A	PZ267715	PZ267716

Abbreviations: y: years; m: months; N/A: not available.

### 3.2 Coproscopical findings

Both Baermann and sedimentation-flotation techniques revealed abundant metastrongyloid larvae (270–320 μm) in faeces from case 1 (Fig. 2). No parasites were detected in the faeces of the surviving male monkey cohabiting with case 1.

**Fig. 2:**
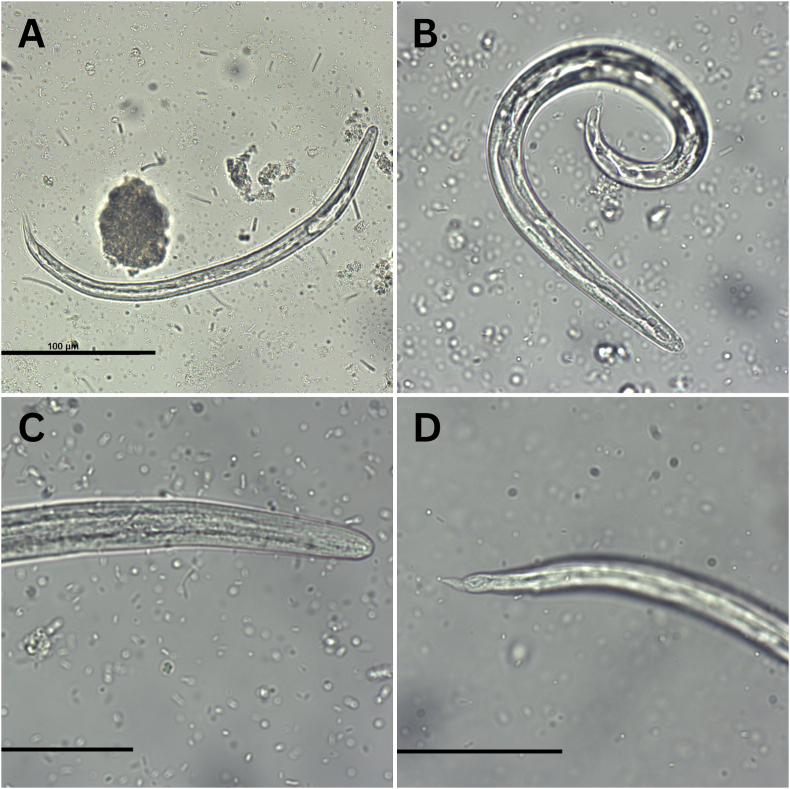
*Angiostrongylus dujardini* larvae recovered from a female *Saguinus oedipus* (case 1) by the Baermann technique. Light microscopic view of slender larvae measuring 270–320 μm in length and 14–18 μm in width, with a transparent cuticle. (A, B) Whole larvae. (C) Slightly tapered anterior end. (D) Posterior end. Scale bars: 100 μm (A), 50 μm (C, D).

### 3.3 PCR and sequencing results

Sequences obtained from faecal larvae and lung tissue (496 bp) of case 1 (PZ267713) were identical to each other and showed 99.6% identity (474/476 bp, 96% query cover, differing in one additional AT-repeat) with a GenBank sequence of *A. dujardini* from a coconut lorikeet (*Trichoglossus haematodus*) from the same zoo (OR268984), and 99.2% identity (492/496 bp, 100% query cover, differing in two additional AT-repeats) with a sequence of *A. dujardini* (GQ181113) from a wood mouse (*Apodemus sylvaticus*) in France. The percentage of identity with sequences from other *Angiostrongylus* species was lower than 82.6%.

Amplification products of the expected size (∼500 bp) were also obtained from four of the retrospectively analysed cases (cases 4–7). In two cases (cases 2 and 3), no amplification was observed, and in one case (case 5) the DNA yield was low, so sequencing was not attempted. Three consensus sequences were obtained in cases 4, 6 and 7 (PZ267714, PZ267715 and PZ267716). The sequence from case 6 was identical to that from case 1. The sequences from case 4 and 7 were identical between them and showed a 100% identity (474/474 bp, 95% query cover) with the sequence of *A. dujardini* from the coconut lorikeet from the same zoo (OR268984).

## 4 Discussion

*Angiostrongylus dujardini* has traditionally been regarded as a parasite primarily affecting rodents (Dróżdż and Doby, 1970; Doby et al., 1971); however, its epidemiology remains poorly understood. This parasite was first described in wood mice (*Apodemus sylvaticus*) and bank voles (*Clethrionomys glareolus*) in France (Doby et al., 1970), followed by further reports in murid and cricetid rodents from Portugal (Doby et al., 1971), Hungary (Mészáros, 1972), Finland (Tenora et al., 1983), and the Iberian Peninsula (Gerrikagoitia et al., 2010). In recent years, infections have been documented in a broader range of host species, with severe cases of angiostrongyliasis reported in zoological institutions in France, Italy and Switzerland involving callitrichid monkeys, suricates and recently, a psittaciform bird (Graille et al., 2015; Eleni et al., 2016; Meister et al., 2024). Clinical signs in affected animals ranged from anorexia, dyspnoea, malaise, and lethargy to death without prior signs.

This case series describes sudden deaths in seven captive cotton-top tamarins (*Saguinus oedipus*) associated with *A. dujardini* infection. The affected animals were aged between 4 months and 6.5 years of both sexes (5 females, 2 males). All individuals were born in European zoological institutions (6 in Switzerland and 1 in Denmark) and were housed in Switzerland, at the same zoo as the above-mentioned coconut lorikeet at the time of death, but not in proximity to the lorikeet's enclosure. Although an aetiological diagnosis was not previously carried out, after the first cases of lungworm infection in the Zoo Basel were registered, a monthly metaphylactic off-label treatment of the cotton-top tamarins was initiated using an oral liquid fenbendazole formulation applied via food. However, sporadic mortalities persisted, possibly due to insufficient uptake via food or inadequate drug efficacy. Peracute mortality was a consistent feature, as five of seven animals were found dead without prior clinical signs. The remaining two monkeys exhibited brief, nonspecific signs of general weakness shortly before death.

Gross pathological examination consistently revealed pulmonary lesions, including multifocal to coalescing, dark red foci, alveolar oedema, and in some cases, serosanguineous thoracic effusion and right-sided cardiac dilation. The cause of death was assumed to be cardio-pulmonary failure.

Histopathology revealed severe pulmonary lesions associated with nematode infection. Lung sections showed widespread vascular and interstitial inflammation with endothelial proliferation, fibrinoid necrosis, and intralesional thrombi, often accompanied by haemorrhage, oedema, and leukocytostasis. Granulomatous inflammation with epithelioid macrophages and multinucleated giant cells was frequenly observed, along with intravascular and intra-alveolar nematode eggs and larvae. These lesions are consistent with both chronic inflammatory processes and acute reactions to the developing parasites. One tamarin showed centrilobular hepatic necrosis, likely due to hypoxia. Although adult parasites were overlooked during routine gross examination in all cases, they were identified histologically in the right ventricle of two animals, with associated myocardial degeneration and inflammation. Therefore, awareness of the possibility of *A. dujardini* infection and careful examination of the heart are required for detection of adult worms (Morgan et al., 2021).

Although the exact source and timing of infections remain unclear, it is likely that the cotton-top tamarins became infected through ingestion of an intermediate or paratenic host, or potentially, via food contaminated with larvae shed with the mucus by infected gastropods as it has been observed in other *Angiostrongylus* species (Spratt, 2015). As the monkeys had access to a naturalistic outdoor enclosure, direct contact with infected gastropod molluscs represents a plausible route of exposure. In addition, the presence of wild rodents in the premises could have served as primary source for environmental contamination with L1. Occasional presence of wild birds in the enclosure, along with observed hunting behaviour by the monkeys, suggests that ingestion of infected birds acting as paratenic hosts could also represent a source of infection. In a French zoo, where fatal *A. dujardini* infections were reported in callitrichid monkeys, adult parasites and L1 shedding were detected in free-ranging rodents (*Apodemus sylvaticus* and *Microtus agrestis*) captured or found dead on the premises (Graille et al., 2015). These findings suggest that the direct presence of wild rodents in zoo environments may serve as a principal source of environmental contamination with this parasite.

In our study, molecular diagnostics confirmed *A. dujardini* infection in four monkeys that died in 2025 (n = 1), 2012 (n = 2), and 2009 (n = 1). The obtained sequences were identical to the sequence previously reported from the fatal angiostrongyliasis case in a coconut lorikeet from the same zoo or differed by only a single AT-repeat. In the three remaining cases, PCR results were negative or weakly positive with low DNA yield (and therefore not sequenced) despite the presence of characteristic histopathological findings. It is noteworthy that the tissue samples from these three cases had been conserved in formalin for six to seven days prior to inclusion in paraffin, while samples from all other cases had been fixed for shorter time periods (one to five days). We assume that the prolonged preservation in the fixative may have accounted for the lack of DNA amplification, as formalin fixation can cause DNA fragmentation and the formation of DNA-protein crosslinks, impairing PCR efficiency (Dietrich et al., 2013).

The findings of this study confirm that *A. dujardini* causes severe pulmonary and cardiovascular pathology in tamarins, leading to peracute mortality with no or only very unspecific clinical signs. This contrasts with the course of natural infections in rodents, which generally develop milder clinical manifestations (Spratt, 2015; Morgan et al., 2021).

In previous studies, L1 were not detected in captive callitrichids using the Baermann technique, although adult worms, verminous pneumonia, and pulmonary endarteritis were observed in these individuals (Graille et al., 2015). Based on the absence of infectious L1 in faecal samples, it was initially hypothesised that these animals might represent accidental or dead-end hosts. However, the present study provides evidence that the parasite can reach sexual maturity, reproduce and be disseminated with the faeces by non-rodent hosts. Nevertheless, the present findings should be interpreted within the context of an anthropogenically created host-parasite association. Cotton-top tamarins are native to South America and would not encounter *A. dujardini* under natural conditions, as the parasite is endemic to Europe. The occurrence of infection in captive callitrichids therefore reflects novel host exposure in zoological environments rather than a naturally occurring host-parasite system. Such host shifts do not necessarily require recent parasite adaptation and may arise through ecological fitting, whereby parasites are able to exploit previously unencountered hosts that possess suitable physiological and ecological characteristics (Hoberg and Brooks, 2008). In this context, although our results demonstrate that *A. dujardini* can reach sexual maturity and produce first-stage larvae in *Saguinus oedipus*, indicating that callitrichids are physiologically competent definitive hosts, these primates should be considered accidental hosts from an evolutionary and biogeographical perspective, as the association occurs outside the parasite's natural host-parasite range. It is further emphasised that experimentally generated or otherwise artificial host-parasite associations may reveal a broader physiological host compatibility than is evident from observations in nature, but do not necessarily reflect the parasite's natural host range or evolutionary history (Poulin and Keeney, 2008). Consistent with this view, our findings demonstrate that *S. oedipus* can function as a competent definitive host for *A. dujardini* under captive conditions, without implying that callitrichids form part of the parasite's natural host spectrum.

## 5 Conclusion

In conclusion, this study confirms the role of cotton-top tamarins (*Saguinus oedipus*) as definitive hosts of *Angiostrongylus dujardini* in zoological settings and highlights the marked pathogenic potential of this parasite in New World primates, characterised by peracute mortality with severe cardiopulmonary lesions. This nematode therefore represents a significant threat to populations of New World primates under human care. Control measures including routine parasitological surveillance, environmental management to reduce exposure to intermediate and paratenic hosts, and consideration of anthelmintic prophylaxis are recommended to mitigate the risk of infection. Furthermore, these findings emphasise the importance of strengthened parasitological monitoring and preventive health management in captive primate colonies.

## Funding source

This research did not receive any specific grant from funding agencies in the public, commercial, or not-for-profit sectors.

## CRediT authorship contribution statement

**Seraina L. Meister:** Data curation, Formal analysis, Methodology, Resources, Writing – original draft, Writing – review & editing. **Christian Wenker:** Data curation, Methodology, Resources, Writing – review & editing. **Michael Rüttener:** Data curation, Methodology, Resources, Writing – review & editing. **Gastón Moré:** Data curation, Methodology, Visualization, Writing – review & editing. **Walter Basso:** Conceptualization, Methodology, Supervision, Validation, Visualization, Writing – original draft, Writing – review & editing.

## Declaration of competing interest

The authors declare that they have no known competing financial interests or personal relationships that could have appeared to influence the work reported in this article.
